# Emergency central aortic repair in acute type A aortic dissection complicated by malperfusion

**DOI:** 10.3389/fsurg.2025.1618755

**Published:** 2025-09-29

**Authors:** Kan-paatib Barnabo Nampoukime, Adeoumi Esperance Monteiro Igwenandji, Youmin Pan, Haihao Wang

**Affiliations:** 1 Division of Cardiothoracic and Vascular Surgery, Tongji Hospital, Tongji Medical College, Huazhong University of Science and Technology, Wuhan, China; 2 Division of Cardiothoracic and Vascular Surgery, Wuhan Tongji Aerospace City Hospital, Wuhan, China

**Keywords:** acute type A aortic dissection, malperfusion, emergency, aortic repair, prognosis

## Abstract

**Objective:**

To assess outcomes of emergency central aortic repair (ECAR) in patients with acute type A aortic dissection (ATAAD) complicated by malperfusion, focusing on in-hospital mortality and long-term survival.

**Methods:**

This retrospective cohort study included 545 ATAAD patients treated surgically at a single center. Patients were stratified into malperfusion (*n* = 149) and non-malperfusion (*n* = 396) groups. Preoperative laboratory parameters, intraoperative strategies, and postoperative outcomes were compared. Kaplan–Meier analysis evaluated long-term survival.

**Results:**

Patients with malperfusion presented with significantly higher D-dimer and creatinine levels and more frequent emergency surgery (73.8% vs. 63.9%, *P* = 0.028). In-hospital mortality was similar between malperfusion and non-malperfusion groups (16.1% vs. 14.1%, *P* = 0.60), but increased with the number of affected organs: 13.3% (single), 18.4% (double), and 30.8% (triple or more). Cardiac and cerebral malperfusion had the highest mortality (40.0%). At 60 months, survival was significantly lower in malperfusion patients (60% vs. 70%, log-rank *P* = 0.00035).

**Conclusion:**

ECAR provides acceptable early survival in ATAAD patients with malperfusion. However, multi-organ involvement significantly worsens both in-hospital and long-term outcomes.

## 1 Introduction

Acute Type A Aortic Dissection (ATAAD) is a life-threatening cardiovascular emergency, accounting for approximately 60%–70% of all aortic dissection cases (1). It is characterized by a tear in the aortic wall, leading to compromised blood flow and, in many cases, malperfusion of multiple organs. Malperfusion occurs in up to 30%–40% of ATAAD cases and is associated with severe ischemia in critical organs such as the kidneys, brain, spinal cord, and heart (2). This complication significantly increases the complexity of patient management and worsens prognosis, as malperfusion leads to higher mortality rates and adds to the surgical challenges (3). The overall in-hospital mortality rate for ATAAD ranges from 20% to 30%, with malperfusion further elevating the risk of death due to organ failure and ischemic injury (4). Current international guidelines by the European Society of Cardiology (ESC, 2014) and the American College of Cardiology/American Heart Association (ACC/AHA, 2022) recommend immediate surgical repair as the standard of care for ATAAD, regardless of malperfusion status. Early diagnosis with computed tomography angiography (CTA), strict blood pressure and heart rate control (target SBP < 120 mmHg, HR < 60 bpm), and rapid surgical intervention are key priorities. For patients with malperfusion, both guidelines recognize that central aortic repair is usually sufficient to restore true lumen flow and resolve end-organ ischemia. However, in cases with severe metabolic acidosis or advanced organ failure, a staged approach with initial endovascular reperfusion may be considered (5, 6). Emergency Central Aortic Repair (ECAR) has emerged as the preferred surgical approach for managing aortic dissection and ischemic complications caused by malperfusion. These recommendations are supported by recent registry data and are aligned with our institutional approach, which prioritizes emergency central aortic repair. However, despite the urgent need for timely intervention, debate continues regarding the optimal surgical strategy and its impact on both short- and long-term survival outcomes (3).

This study aims to evaluate the efficacy and outcomes of ECAR in ATAAD patients with malperfusion, focusing on in-hospital mortality and long-term survival rates.

## 2 Patients and method

This retrospective study included patients who underwent surgical treatment for ATAAD at Tongji Hospital between January 2019 and November 2019, and from January 2021 to December 2022. A total of 561 consecutive patients were initially screened.

Exclusion criteria were as follows: one patient who died due to preoperative aortic rupture, four patients who could not be weaned off cardiopulmonary bypass intraoperatively, three patients with incomplete clinical data, and eight patients who underwent early organ reperfusion before central aortic repair. In total, 16 patients were excluded, resulting in a final study cohort of 545 patients.

The included patients were categorized into two groups based on the presence or absence of malperfusion at presentation: the non-malperfusion group (*n* = 396) and the malperfusion group (*n* = 149).

### 2.1 Definitions

#### 2.1.1 Coronary malperfusion

Identified by coronary malperfusion on computed tomography angiography (CTA), accompanied by elevated serum troponin levels (>34.2 pg/ml), and/or ischemic changes on electrocardiography (ECG), and/or myocardial ischemia confirmed by echocardiography. Visceral Malperfusion: Renal Malperfusion: Confirmed by CTA evidence of renal artery malperfusion, along with elevated serum creatinine levels and decreased estimated glomerular filtration rate (eGFR).Celiac and/or Mesenteric Malperfusion (abdominal malperfusion): Confirmed by CTA evidence of celiac or mesenteric artery malperfusion, accompanied by increased serum uric acid levels and/or elevated liver enzymes. Peripheral Malperfusion: Cerebral Malperfusion: Characterized by neurological symptoms such as headache, dizziness, or altered consciousness, with CTA confirmation of blood flow obstruction in the supra-aortic vessels. Limb Malperfusion: Identified by CTA evidence of blood flow obstruction in the iliac or femoral arteries, accompanied by clinical signs such as absent peripheral pulses, numbness, and loss of motor function (7, 8).

## 3 Surgical procedures

The primary surgical approach for ATAAD at our institution is ECAR, performed via median sternotomy under cardiopulmonary bypass (CPB) and moderate to deep hypothermic circulatory arrest, depending on the complexity and extent of dissection (target bladder temperature 20–26°C). Arterial cannulation is achieved via the right axillary artery, femoral artery, direct aortic cannulation, or a combination thereof, with left heart venting via the right superior pulmonary vein. Myocardial protection is ensured with cold blood cardioplegia delivered either antegrade or retrograde. Circulatory arrest is initiated to facilitate open distal anastomosis, and selective antegrade cerebral perfusion is employed when extended arch repair is necessary.

The choice of surgical technique is determined by tear location and the extent of aortic involvement. In cases confined to the ascending aorta, limited replacement of the ascending aorta or hemiarch is performed. If the dissection extends into or originates from the aortic arch or involves the descending thoracic aorta, total arch replacement with frozen elephant trunk (FET) implantation is carried out using Sun's procedure.

### 3.1 Aortic root and valve management

Aortic valve preservation is attempted when feasible. However, in patients with root dilatation, annuloaortic ectasia, or dissection involving the sinus of Valsalva, a composite graft replacement (Bentall procedure) is performed. After mobilizing the aortic root and preparing coronary buttons, the native valve is excised. A mechanical valved conduit is implanted, secured to the annulus with 2-0 pledgeted U-sutures. The coronary ostia are reimplanted onto the graft using the modified button technique. The distal anastomosis is reinforced with felt strips, and de-airing is meticulously performed before aortic unclamping.

### 3.2 Total arch replacement (sun’s procedure)

In patients with arch involvement or malperfusion affecting distal organs, the Sun's procedure is implemented. Following systemic cooling to 25°C, the aortic arch is opened after clamping the supra-aortic vessels. A self-expanding stented graft (FET) is deployed into the descending aorta, followed by distal anastomosis to a tetrafurcated Dacron graft that replaces the aortic arch. Lower body perfusion is resumed via a side branch of the graft. The supra-aortic vessels are then reconstructed sequentially (innominate, left carotid, and left subclavian arteries). The proximal end of the arch graft is then anastomosed to the distal end of the previously implanted ascending graft or valved conduit.

In some patients with extensive aortic disease or evidence of persistent or residual malperfusion, additional thoracic endovascular aortic repair (TEVAR) is required. TEVAR may be performed intraoperatively in a hybrid operating room, particularly when the dissection extends into the abdominal aorta, or when there is involvement of branch vessels such as the renal, mesenteric, or iliac arteries, and where true lumen compression or occlusion persists despite central repair.

Patients presenting with preoperative malperfusion symptoms—such as abdominal pain (mesenteric ischemia), decreased urine output (renal ischemia), or lower limb ischemia (femoral artery involvement)—are closely monitored postoperatively. In stable patients, if postoperative computed tomography angiography (CTA) reveals ongoing malperfusion or inadequate re-expansion of the true lumen at distal branch points, staged TEVAR or fenestration/stenting of the involved arteries is performed. This approach allows targeted restoration of end-organ perfusion, particularly in the setting of static or dynamic obstruction that is not resolved by proximal aortic repair alone.

The integration of open and endovascular techniques through the hybrid strategy has become a key component of our institutional protocol, enabling individualized and anatomy-driven treatment for patients with complex aortic dissection and malperfusion syndromes. This approach enhances procedural flexibility, reduces time to reperfusion, and contributes to improved postoperative recovery and organ function preservation.

### 3.3 Statistical analysis

Descriptive statistics were calculated for both continuous and categorical variables. Continuous variables were reported as mean ± standard deviation (SD) and median where appropriate, while categorical variables were presented as counts and percentages. Group comparisons between patients with and without malperfusion were performed using Welch's two-sample t-test for continuous variables, Pearson's chi-square test for categorical variables with expected cell counts ≥5, and Fisher's exact test when any expected count was <5. Data on specific causes of death, post-discharge revascularization procedures, and rehospitalizations were collected where available. However, systematic cause-of-death documentation was not consistently present in the medical records, and no standardized registry for post-discharge interventions was maintained. For survival analysis, all patients were followed up via telephone contact, with the last follow-up date recorded as October 15, 2024. The follow-up completion rate was 95.6% (521 out of 545 patients), and survival status was confirmed in all cases with complete follow-up. Kaplan–Meier curves were generated to compare survival between groups, and the log-rank test was used to assess statistical significance. Missing data were minimal (<5% for all variables). No imputation was performed; analyses were conducted on available data. Variables with missing values were excluded pairwise in bivariate analyses to maximize data use without introducing bias. All statistical analyses were conducted at a two-sided significance level of 0.05 using R version 4.4.2.

## 4 Results

Among 545 ATAAD patients, 27.3% had malperfusion. Compared with non-malperfusion patients, this group showed significantly higher markers of ischemia (D-dimer, LDH, troponin, creatinine) and lower fibrinogen and eGFR (Table 1).

**Table 1:** Preoperative characteristics.

Variables	Overall	Malperfusion	Non malperfusion	*P*-value
Male, *n* (%)	427 (78.3%)	122 (81.9%)	305 (77.0%)	0.20
Age (years, mean ± SD)	51.6 (11.5)	51.7 (11.3)	51.6 (11.5)	>0.90
BMI (kg/m^2^, mean ± SD)	25.5 (4.0)	25.6 (3.7)	25.4 (4.1)	0.70
Smoking history, *n* (%)	132 (24.2%)	36 (24.2%)	96 (24.2%)	>0.90
Diabetes history, *n* (%)	42 (7.7%)	11 (7.4%)	31 (7.8%)	0.90
Coronary artery disease history, *n* (%)	35 (6.4%)	12 (8.1%)	23 (5.8%)	0.30
Hypertension history, *n* (%)	329 (60.4%)	90 (60.4%)	239 (60.4%)	0.90
Pericardial effusion, *n* (%)	152 (27.9%)	43 (28.9%)	109 (27.5%)	0.80
Previous cardiac surgery, *n* (%)	10 (1.8%)	2 (1.3%)	8 (2.0%)	0.70
History of TEVAR, *n* (%)	9 (1.7%)	0 (0.0%)	9 (2.3%)	0.12
Marfan syndrome, *n* (%)	8 (1.5%)	2 (1.3%)	6 (1.5%)	>0.90
LVEF (%)	57.9 (6.9)	57.6 (7.2)	58.1 (6.8)	0.50
Aortic root diameter (mm)	38.7 (9.6)	39.1 (10.6)	38.5 (9.3)	0.50
D-dimer (mg/L)	11.3 (9.5)	13.5 (9.8)	10.5 (9.3)	0.001
Fibrinogen (g/L)	2.9 (1.8)	2.5 (1.2)	3.1 (2.0)	<0.001
ALT (U/L)	44.8 (151.5)	57.0 (198.4)	40.3 (129.5)	0.30
AST (U/L)	66.3 (253.2)	97.3 (304.3)	54.7 (230.4)	0.12
eGFR (ml/min/1.73 m^2^)	79.0 (32.3)	66.0 (35.3)	83.9 (29.6)	<0.001
Uric acid (mg/L)	381.6 (144.9)	429.2 (144.5)	363.7 (141.2)	<0.001
Cholesterol (g/L)	4.2 (3.4)	4.4 (5.2)	4.1 (2.4)	0.50
CK (U/L)	312.5 (1,448.6)	515.8 (1,856.4)	236.0 (1,255.9)	0.091
LDH (μ/L)	295.2 (217.9)	355.1 (263.0)	272.7 (194.0)	<0.001
Creatinine (µmol/L)	115.6 (122.3)	151.0 (165.8)	102.3 (98.2)	<0.001
Troponin (ng/ml)	1,528.0 (6,909.1)	3,694.2 (11,175.4)	712.9 (4,063.4)	0.002
CK-MB (U/L)	6.0 (20.3)	11.7 (34.6)	3.8 (9.9)	0.007
NT-pro BNP (pg/ml)	1,027.7 (3,441.6)	1,289.5 (4,138.9)	928.7 (3,138.2)	0.3
Myoglobin (ng/ml)	177.4 (294.6)	274.3 (390.0)	141.0 (240.1)	<0.001
Preoperative Intubation, *n* (%)	260 (47.7%)	79 (53.0%)	181 (45.7%)	0.13
Aortic Valve Regurgitation, *n* (%)	–	–	–	0.80
No	370 (67.9%)	104 (69.8%)	266 (67.2%)	–
Grade I	23 (4.2%)	5 (3.4%)	18 (4.5%)	–
Grade II	152 (27.9%)	40 (26.8%)	112 (28.3%)	–
Extension of aortic dissection, *n* (%)	–	–	–	0.13
Aortic arch	104 (19.1%)	22 (14.8%)	82 (20.7%)	–
Descending aorta	178 (32.7%)	57 (38.3%)	121 (30.6%)	–
Aortic arch vessels	263 (48.3%)	70 (47.0%)	193 (48.7%)	–
Surgery performed within 24 h of symptom onset	363 (66.6%)	110 (73.8%)	253 (63.9%)	0.028

Surgical procedures were more complex in the malperfusion group, reflected by longer cardiopulmonary bypass times and a greater need for concomitant TEVAR (Table 2). Despite greater surgical complexity, early mortality did not differ significantly between groups. Malperfusion, however, was associated with a higher incidence of postoperative acute kidney injury and greater use of continuous renal replacement therapy (CRRT) (Table 3). CRRT was often required in patients with severe electrolyte imbalance, volume overload, or metabolic acidosis necessitating dialysis support. Circulatory failure was observed in 8 (5.4%) patients with malperfusion and 20 (5.1%) without malperfusion, typically occurring intraoperatively or within the first 24 h postoperatively (Table 2). Management included high-dose inotropic support and, in some cases, ECMO.

**Table 2:** Intraoperative characteristics.

Variables	Overall (*n* = 545)	MPF (*n* = 149)	Non-MPF (*n* = 396)	*P*-value
Arterial Cannulation, *n* (%)				
Subclavian + Femoral artery	233 (42.8%)	61 (40.9%)	172 (43.4%)	0.60
Femoral artery	93 (17.1%)	26 (17.4%)	67 (16.9%)	0.90
Subclavian artery	204 (37.4%)	58 (38.9%)	146 (36.9%)	0.70
Aortic artery	15 (2.8%)	4 (2.7%)	11 (2.8%)	>0.90
Cerebral perfusion	247 (45.3%)	62 (41.6%)	185 (46.7%)	0.30
Surgical time, (minutes)	534.8 (122.8)	547.4 (123.5)	530.0 (122.4)	0.14
CPB Time, (minutes)	237.8 (71.9)	250.6 (75.3)	233.0 (70.1)	0.014
Aortic clamping Time	122.9 (36.9)	128.0 (38.7)	120.9 (36.1)	0.052
Intraoperative temperature (°C)	26.1 (2.6)	26.1 (2.5)	26.2 (2.7)	0.70
Ascending aortic replacement, *n* (%)	132 (24.2%)	34 (22.8%)	98 (24.7%)	0.60
Aortic arch replacement	413 (75.8%)	115 (77.2%)	298 (75.3%)	0.60
Aortic repair + TEVAR	18 (3.3%)	13 (8.7%)	5 (1.3%)	<0.001
Aortic repair + CABG	55 (10.1%)	19 (12.8%)	36 (9.1%)	0.20
Circulatory failure, *n* (%)	28 (5.1%)	8 (5.4%)	20 (5.1%)	0.90

**Table 3:** Postoperative characteristics.

Variable	Overall	Malperfusion	Non malperfusion	*P*-value
ICU stay (days)	11.6 (11.6)	12.8 (13.1)	11.2 (11.0)	0.20
Hospital stay (days)	23.6 (15.0)	25.0 (18.7)	23.1 (13.3)	0.30
Re-exploration, *n* (%)	28 (5.1%)	7 (4.7%)	21 (5.3%)	0.80
ECMO, *n* (%)	16 (2.9%)	6 (4.0%)	10 (2.5%)	0.40
CRRT, *n* (%)	114 (20.9%)	45 (30.2%)	69 (17.4%)	0.001
Tracheotomy, *n* (%)	85 (15.6%)	28 (18.8%)	57 (14.4%)	0.20
In-hospital mortality, *n* (%)	80 (14.7%)	24 (16.1%)	56 (14.1%)	0.60
MODS, *n* (%)	12 (2.2%)	4 (2.7%)	8 (2.0%)	0.70
Coma, *n* (%)	33 (6.1%)	13 (8.7%)	20 (5.1%)	0.11
Paralysis, *n* (%)	18 (3.3%)	8 (5.4%)	10 (2.5%)	0.11
Stroke, *n* (%)	17 (3.1%)	7 (4.7%)	10 (2.5%)	0.30
Pulmonary infection, *n* (%)	90 (16.5%)	25 (16.8%)	65 (16.4%)	>0.90
Gastrointestinal bleeding, *n* (%)	24 (4.4%)	8 (5.4%)	16 (4.0%)	0.50
AKI, *n* (%)	26 (4.8%)	15 (10.1%)	11 (2.8%)	<0.001

Acute Kidney Injury.

Outcomes varied markedly with the extent and type of malperfusion (Table 4). Mortality rose proportionally with the extent of organ involvement. Cardiac and cerebral malperfusion, particularly in combination, carried the highest mortality and complication burden. The overall mortality rate reflects the weighted average across all categories; subgroup percentages are calculated within each category and therefore do not sum to the total. Detailed causes of in-hospital death were not systematically documented in all cases and could not be reliably classified; therefore, only overall mortality is reported. No additional post-discharge revascularization procedures, endovascular interventions, or rehospitalizations could be analyzed due to incomplete data collection in this retrospective cohort.

**Table 4:** Comprehensive malperfusion subgroup analysis.

Malperfusion type	Patients (*n*)	Deaths (*n*)	Mortality (%)	Mean ICU stay (days)	Key complications
Single-organ malperfusion	98	13	13.3%	9.2	
Cerebral	10	2	20.0%	12.1	Stroke
Renal	45	5	11.1%	10.8	AKI
Abdominal	25	3	12.0%	7.5	GI bleeding
Cardiac	12	2	16.7%	15.3	MODS
Limb	6	1	16.7%	5.2	Ischemia
Double-organ malperfusion	38	7	18.4%	13.6	
Cerebral + Renal	8	2	25.0%	14.8	Stroke + AKI
Cardiac + Renal	12	2	16.7%	16.2	MODS + AKI
Abdominal + Renal	10	1	10.0%	11.4	GI bleeding + AKI
Cardiac + Cerebral	5	2	40.0%	18.6	Stroke + MODS
Cerebral + Limb	3	0	0.0%	9.0	Ischemia
Triple or more organs	13	4	30.8%	19.4	
Cardiac + Renal + Cerebral	6	2	33.3%	21.2	MODS, Stroke,
Abdominal + Renal + Limb	4	1	25.0%	16.8	GI bleed
More organs	3	1	33.3%	17.5	MODS
Total	149	24	16.1%	—	—

**Figure 1:**
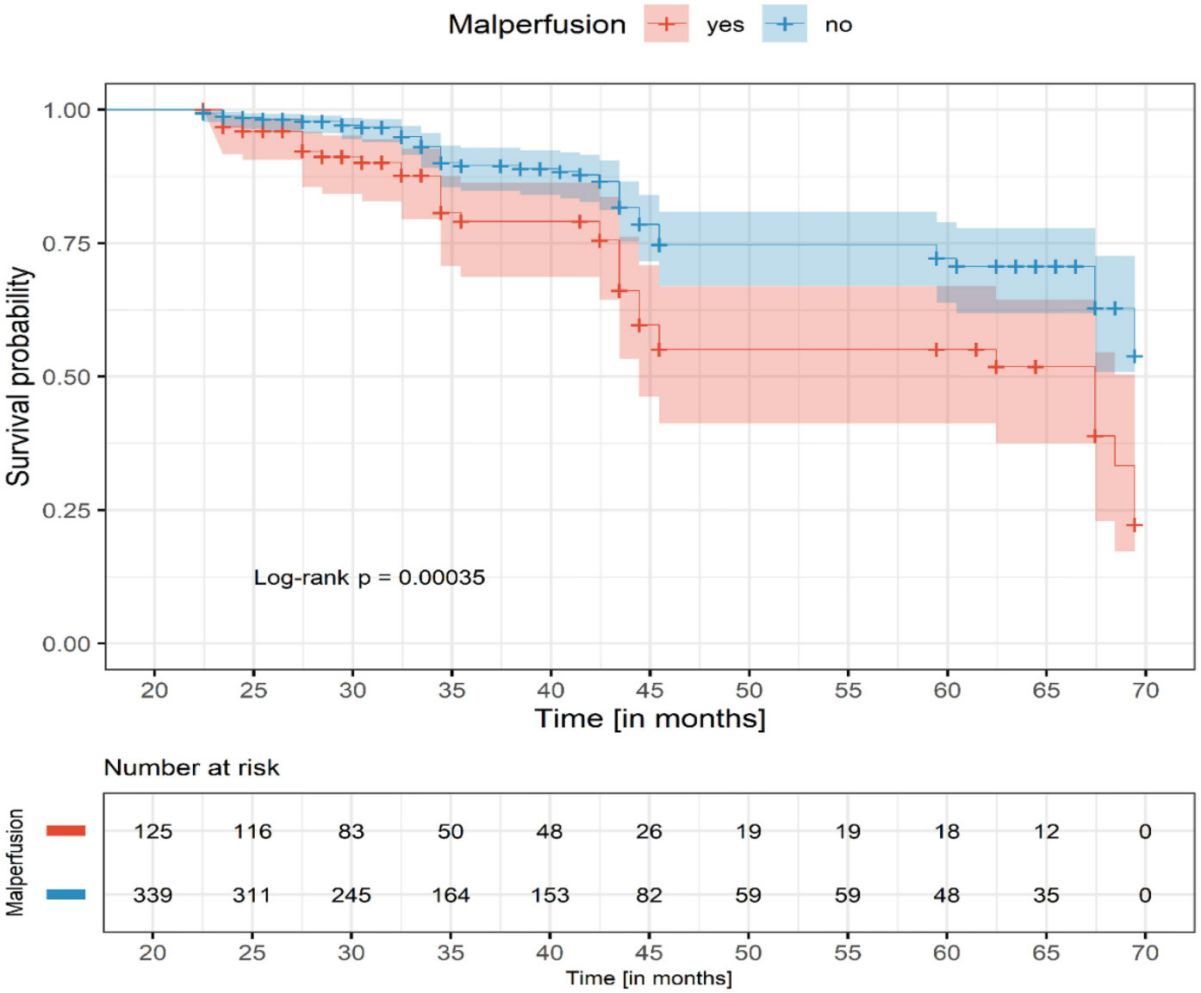
Kaplan–Meier survival curve.

Long-term survival was significantly lower in patients with malperfusion. Kaplan–Meier analysis (Figure 1) demonstrated early similarity but worse long-term survival in patients with malperfusion.

## 5 Discussion

In this study of 545 patients with ATAAD, malperfusion was present in 27.3% of cases and was associated with greater surgical complexity and more frequent postoperative complications, particularly acute kidney injury. Despite this, early mortality after ECAR was similar between malperfusion and non-malperfusion groups (16.1% vs. 14.1%). However, mortality increased proportionally with the number of affected organs, reaching 30.8% in patients with ≥3 territories involved. Long-term survival was significantly worse in the malperfusion group, with 60-month survival of 60% compared to 70% in those without malperfusion (Table 4; Figure 1).

Patients with malperfusion demonstrated distinct laboratory profiles that reflect the severity of systemic ischemia and coagulopathy. Elevated D-dimer and reduced fibrinogen levels indicate consumptive coagulopathy and inflammation, while impaired renal function and elevated cardiac biomarkers (troponin, CK-MB) suggest subclinical organ ischemia (9–11). These laboratory findings, rather than traditional cardiovascular risk factors, provide a more accurate indication of malperfusion severity. They underscore the need for prompt intervention with ECAR to restore perfusion and prevent irreversible organ damage. Together with imaging, these biomarkers form the cornerstone of preoperative risk stratification and guide timely surgical decision-making (12, 13).

### 5.1 Management of ATAAD with malperfusion

The management of ATAAD complicated by malperfusion is influenced by multiple factors, including surgeon expertise, institutional resources, patient risk profile, and anatomical considerations. The optimal strategy remains controversial. In patients with malperfusion syndrome, endovascular fenestration or stenting before definitive central aortic repair has gained acceptance in selected cases, as it can promptly restore branch vessel flow and stabilize the patient prior to open repair (14). The “reperfusion-first” strategy initial endovascular revascularization followed by delayed aortic repair has been increasingly adopted, aiming to reduce metabolic stress and mitigate ischemia–reperfusion injury (15). For cerebral malperfusion, this approach has been associated with lower early mortality compared to a central repair-first strategy and a reduction in neurological complications (16).

However, emerging evidence supports ECAR as an effective means to decompress the false lumen, re-establish true lumen flow, and restore organ perfusion, potentially avoiding irreversible ischemic damage (17). A time-dependent strategy has also been proposed for mesenteric malperfusion, with immediate central repair for patients presenting within 6 h of symptom onset, and reperfusion-first intervention for those beyond 6 h; this approach demonstrated a significant mortality benefit (18.5% vs. 54.6%) (18).

Our institutional protocol favors immediate ECAR without delay, with the goal of minimizing time to organ reperfusion. In our cohort, patients with malperfusion underwent longer cardiopulmonary bypass and more frequent adjunctive endovascular procedures, reflecting the added technical complexity of their surgery. These findings underscore the importance of surgical readiness, hybrid operating room capability, and coordinated multidisciplinary management. Ultimately, optimal outcomes depend on early recognition of malperfusion, individualized decision-making, and integrated perioperative care.

### 5.2 Patterns of organ involvement

Malperfusion complicates 10%–33% of acute aortic dissection cases (19). Goel et al. reported extremity malperfusion as the most common presentation, whereas in our cohort renal involvement predominated (20).

Mortality risk differs by organ system, with mesenteric, cardiac, and cerebral malperfusion consistently reported as the most lethal patterns, and cerebral and cardiac involvement highlighted as dominant predictors of early death (21). In our cohort, renal malperfusion was the most frequent presentation, whereas cerebral and cardiac involvement carried the highest relative mortality risk, findings consistent with prior reports. Our data confirmed a stepwise increase in risk with multi-organ malperfusion (Table 4). These findings are consistent with prior evidence demonstrating a dose–response relationship between malperfusion burden and mortality. Czerny et al. reported markedly reduced survival with multi-territory ischemia (18). Taken together, these data support ECAR as a feasible first-line strategy in ATAAD with malperfusion, while underscoring those outcomes are strongly modulated by the territory and burden of ischemia. Although renal malperfusion was most frequent in our cohort, excess risk concentrated in cerebral, coronary, and mesenteric involvement and rose stepwise with multi-organ malperfusion (single→double→≥3 territories). Thus, ECAR should be paired with territory-specific adjuncts (e.g., rapid myocardial/cerebral protection, early assessment and selective endovascular or surgical reperfusion for mesenteric ischemia) and aggressive early postoperative monitoring, as the early hazard is greatest in these phenotypes. Overall, our findings favor prompt central aortic repair to restore true-lumen flow, with selective staged or concomitant reperfusion tailored to high-risk organ beds and to the cumulative malperfusion load.

### 5.3 Postoperative complications

Postoperative Acute kidney Injury were notably prevalent among renal malperfusion patients requiring more use of CRRT, compared to other subgroups. Need for CRRT likely reflects both preexisting ischemia and anatomical factors such as renal artery involvement and persistent false lumen compression (22, 23), as well as postoperatory systemic derangements including metabolic acidosis, electrolyte imbalances, and fluid overload in critically ill patients. Although complications like stroke, coma, and MODS showed variable incidence across groups, the kidneys appear particularly vulnerable due to their perfusion characteristics. These findings emphasize the importance of early renal function monitoring and timely initiation of CRRT to prevent progression acute kidney injury to chronic renal dysfunction.

### 5.4 In-hospital mortality and long-term survival

Early mortality was comparable between groups (Table 3), consistent with prior reports. while Nicholas J. Goel et al. observed significantly greater mortality in the malperfusion group (26.8% vs. 13.6%; *P* < 0.001) suggesting that advances in surgical technique and perioperative management have narrowed the early survival gap (20, 24).

In the study by Wang et al., patients with branch vessel involvement and those with organ malperfusion demonstrated lower two-year survival compared with those without (25). Similarly, survival curves diverged during follow-up, with malperfusion patients showing worse outcomes (Figure 1). Persistent organ dysfunction especially renal and neurological likely contributes to this decline. These findings are consistent with data from the International Registry of Acute Aortic Dissection, which also report higher early mortality and poorer long-term survival in malperfusion patients (4, 19).

In contrast, other studies have suggested a more favorable long-term outlook for this population. For instance, Chiu et al. found that mid-term mortality following immediate surgical repair was comparable between ATAAD patients with and without malperfusion (26). Similarly, Kawahito et al. reported favorable long-term outcomes among operative survivors, implying that once patients survive the acute phase, their prognosis may parallel that of patients without malperfusion (3). These discrepancies may be attributed to differences in study populations, definitions of malperfusion, surgical timing, or postoperative surveillance practices.

These findings suggest that while ECAR has helped mitigate early mortality in ATAAD patients with malperfusion, the consequences of early ischemic injury may continue to shape long-term outcomes. Prospective, multicenter studies are needed to clarify these variations and to better identify patients at risk for late complications despite successful surgical intervention.

### 5.5 Study limitations

Several limitations should be acknowledged. First, this was a retrospective, single-center study, which may limit generalizability and introduce selection bias. Second, detailed cause-of-death information was not systematically documented, and post-discharge data on secondary interventions such as staged TEVAR, additional revascularization, or rehospitalizations were incomplete, limiting the ability to assess their impact on long-term outcomes. Third, organ function recovery was not longitudinally tracked, particularly renal, cerebral, or myocardial status after discharge. Additionally, perfusion adequacy was not quantified using advanced imaging modalities (e.g., CT perfusion or intraoperative Doppler), which could enhance future risk stratification. Finally, quality-of-life outcomes were not assessed, despite their growing importance in ATAAD survivorship. Prospective, multicenter studies with comprehensive follow-up are needed to address these gaps and refine risk stratification.

## 6 Conclusion

In acute type A aortic dissection, malperfusion significantly affects preoperative status, increases surgical complexity, and compromises long-term outcomes. ECAR appears effective in stabilizing patients and achieving acceptable early survival, even in those with malperfusion.

However, the extent and pattern of malperfusion play a critical role in prognosis. Patients with multiple organ involvement, particularly those with combined cardiac and cerebral malperfusion, are at markedly higher risk of mortality and complications. These findings underscore the importance of early recognition, individualized surgical strategies, and proactive postoperative monitoring.

A comprehensive approach combining imaging, biomarkers, and timely surgical intervention is essential to improve both early and late outcomes in this high-risk population.

## Data Availability

The raw data supporting the conclusions of this article will be made available by the authors, without undue reservation.
